# Supplementary figures and images for: Effects of HSP70 chaperones Ssa1 and Ssa2 on Ste5 scaffold and the mating mitogen-activated protein kinase (MAPK) pathway in Saccharomyces cerevisiae
Source: PLoS One. 2023 Oct 18;18(10):e0289339. doi: 10.1371/journal.pone.0289339 (PMC10584130; doi:10.1371/journal.pone.0289339)

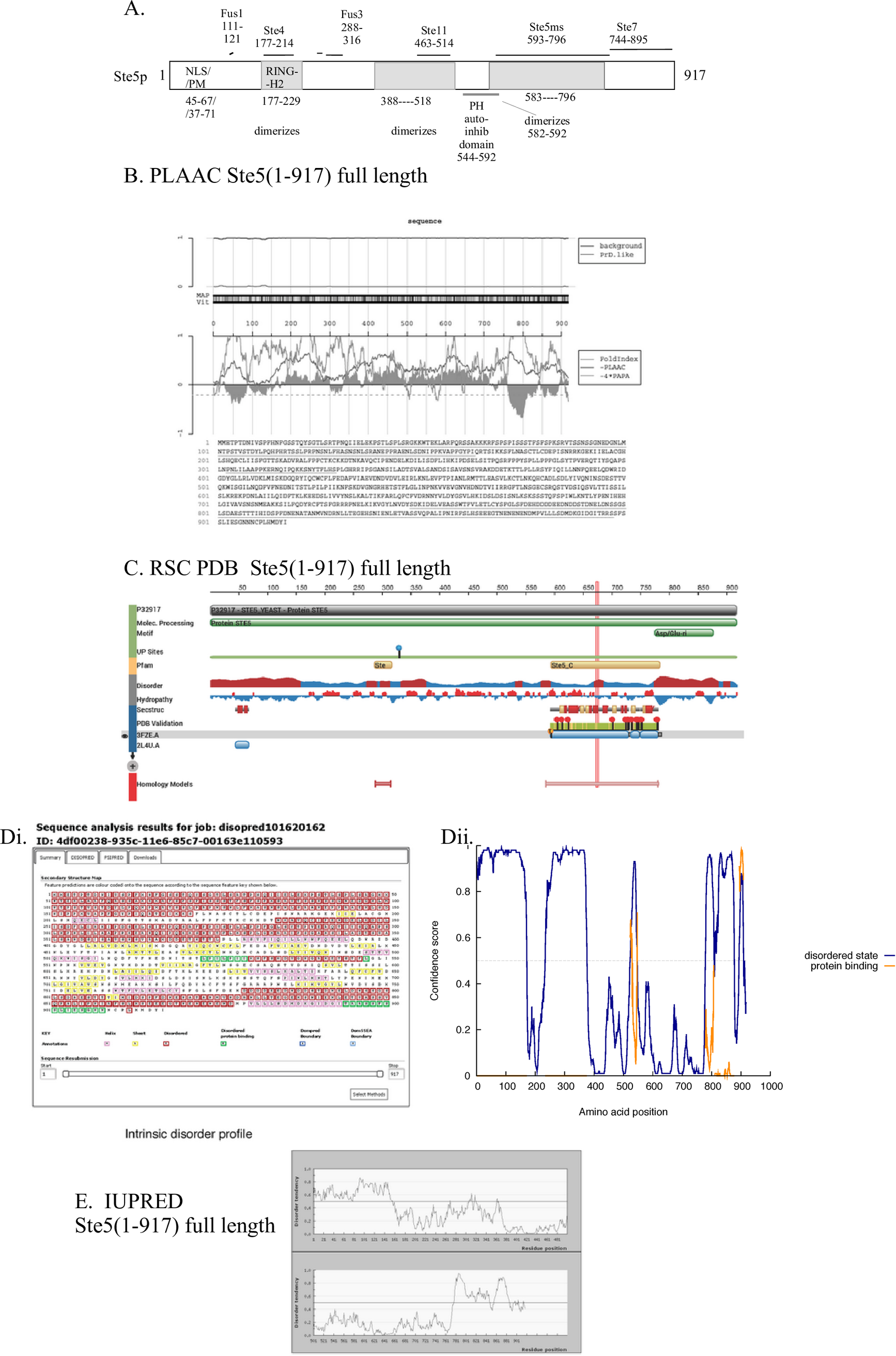

Supplement: S1 Fig — (TIF) [file pone.0289339.s001.tif]

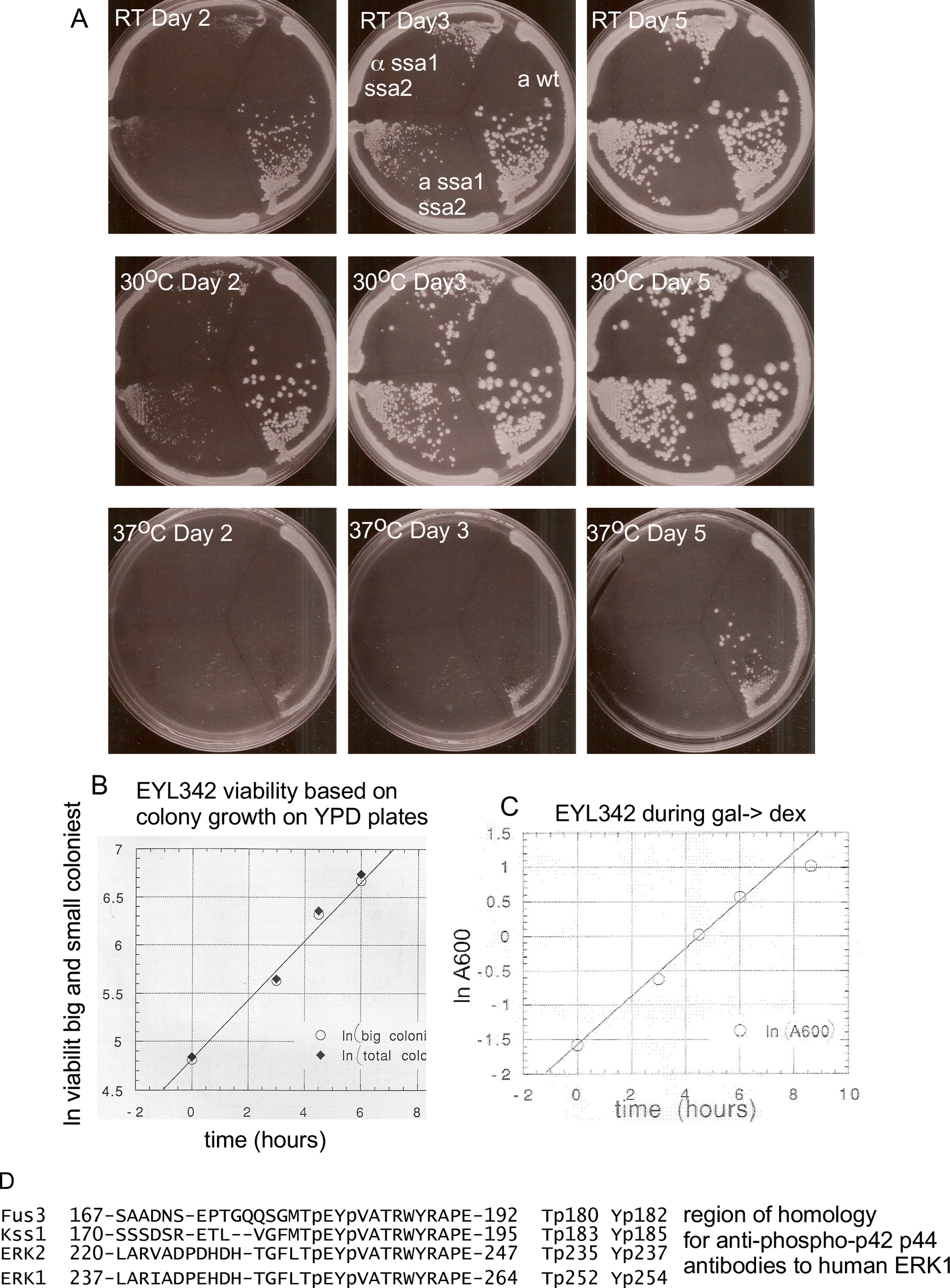

Supplement: S2 Fig — (TIF) [file pone.0289339.s002.tif]

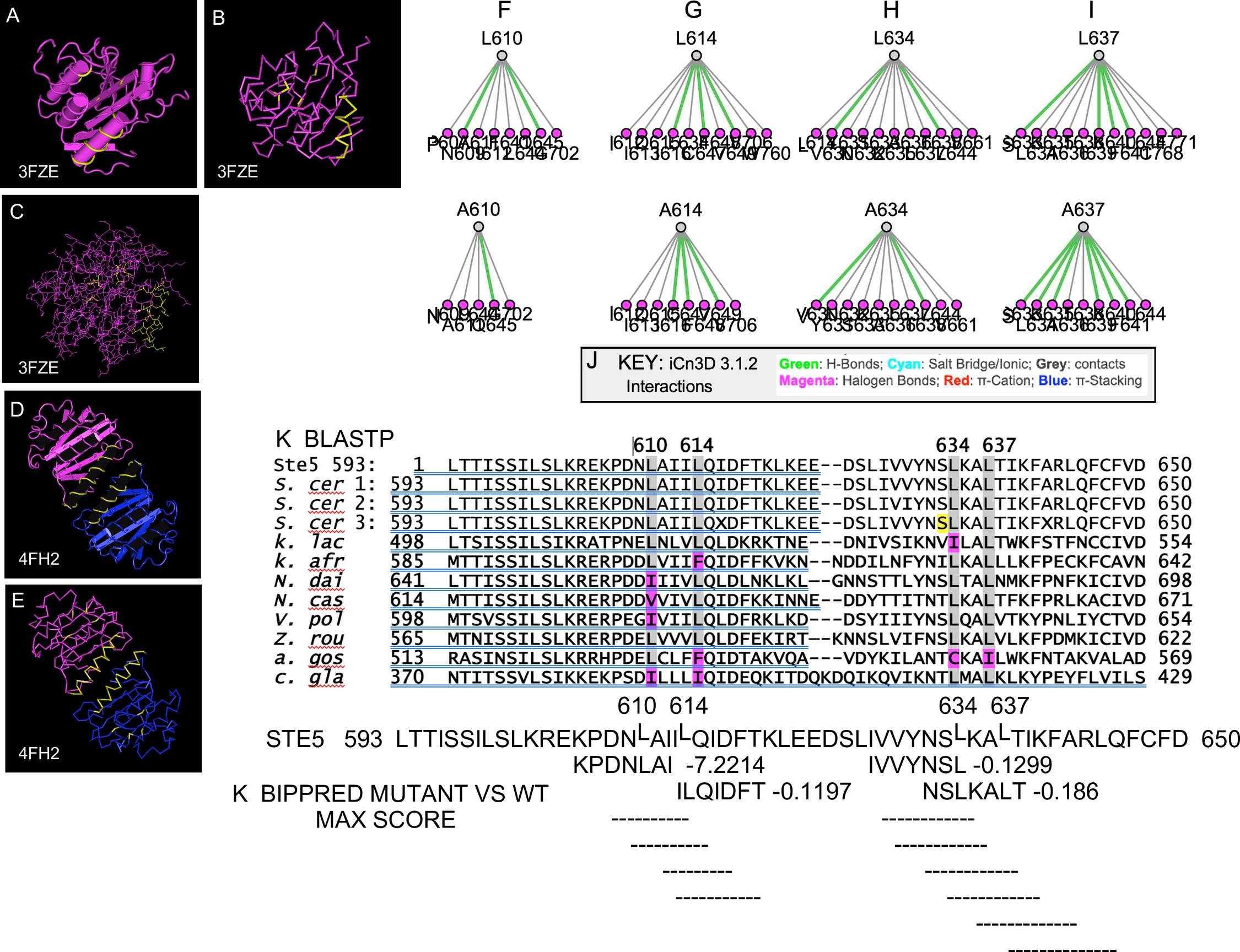

Supplement: S3 Fig — (TIF) [file pone.0289339.s003.tif]

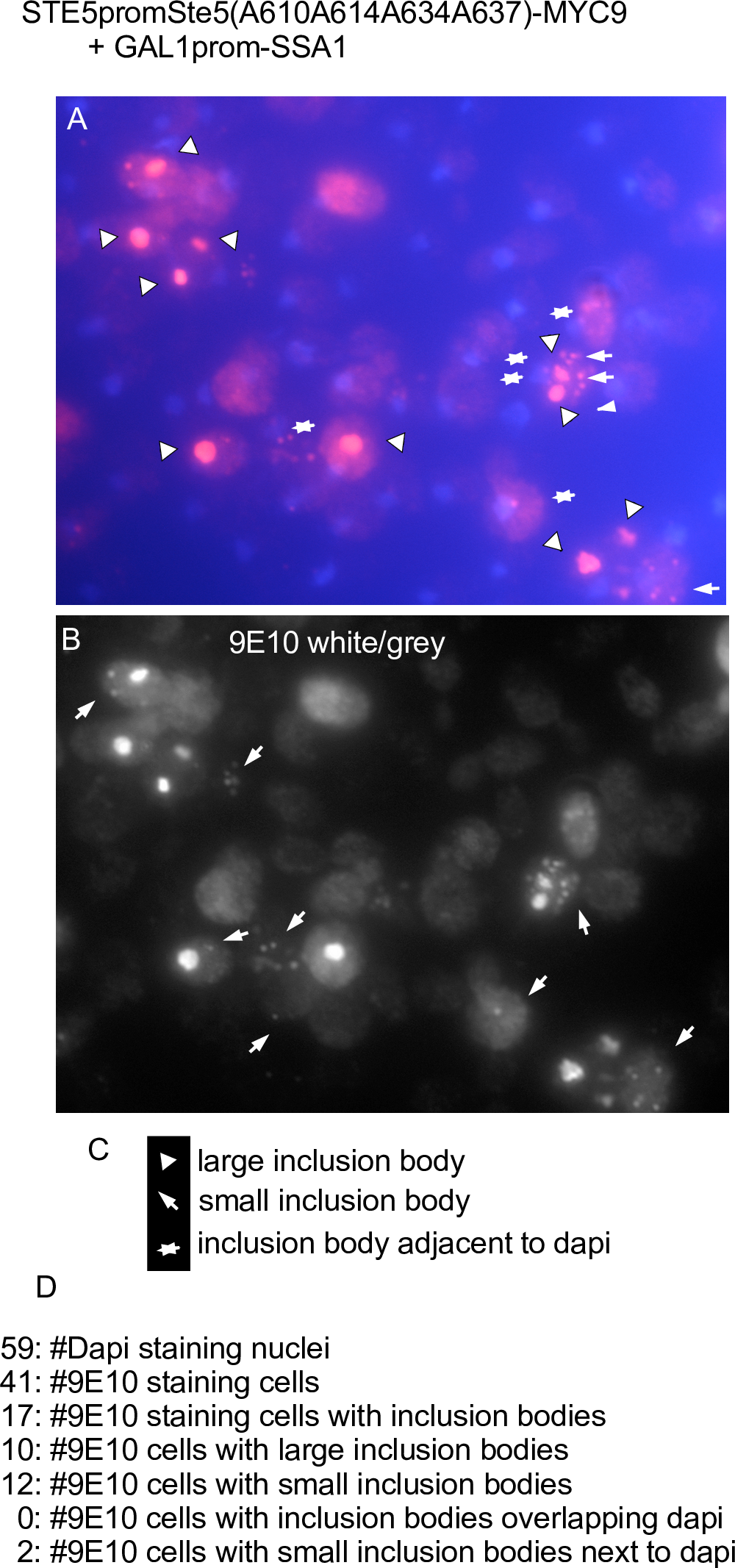

Supplement: S4 Fig — (TIF) [file pone.0289339.s004.tif]

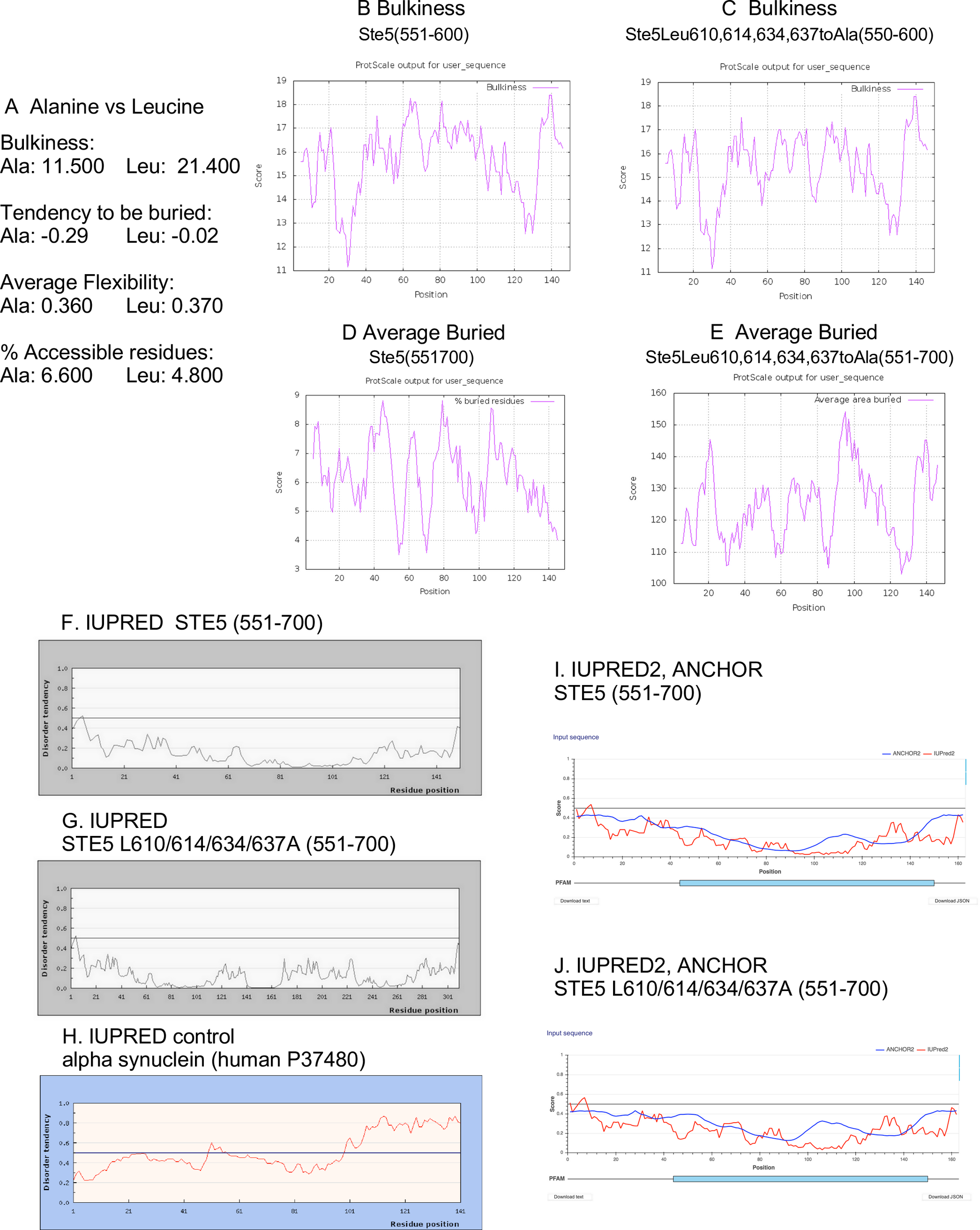

Supplement: S5 Fig — (TIF) [file pone.0289339.s005.tif]

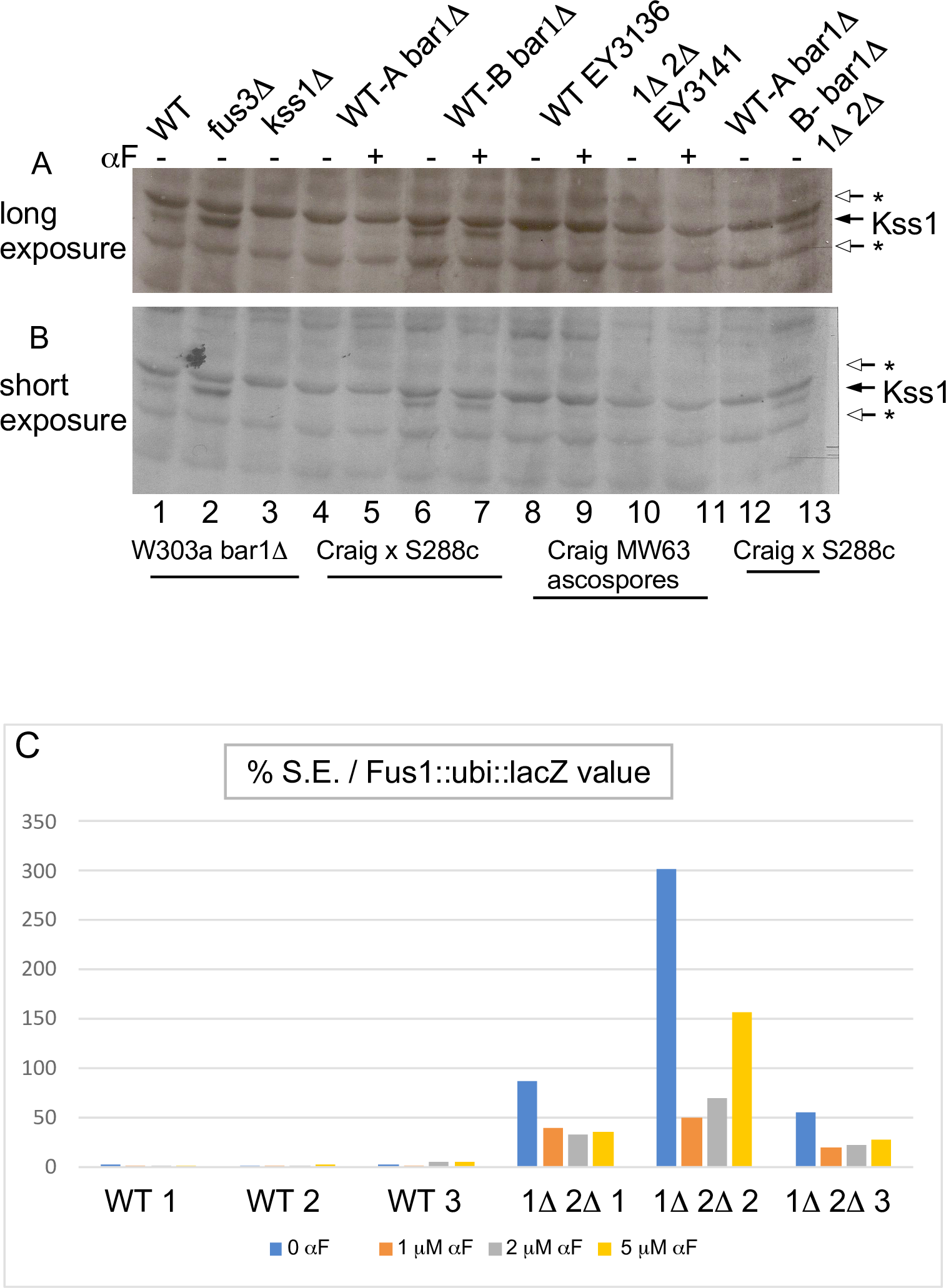

Supplement: S6 Fig — (TIF) [file pone.0289339.s006.tif]

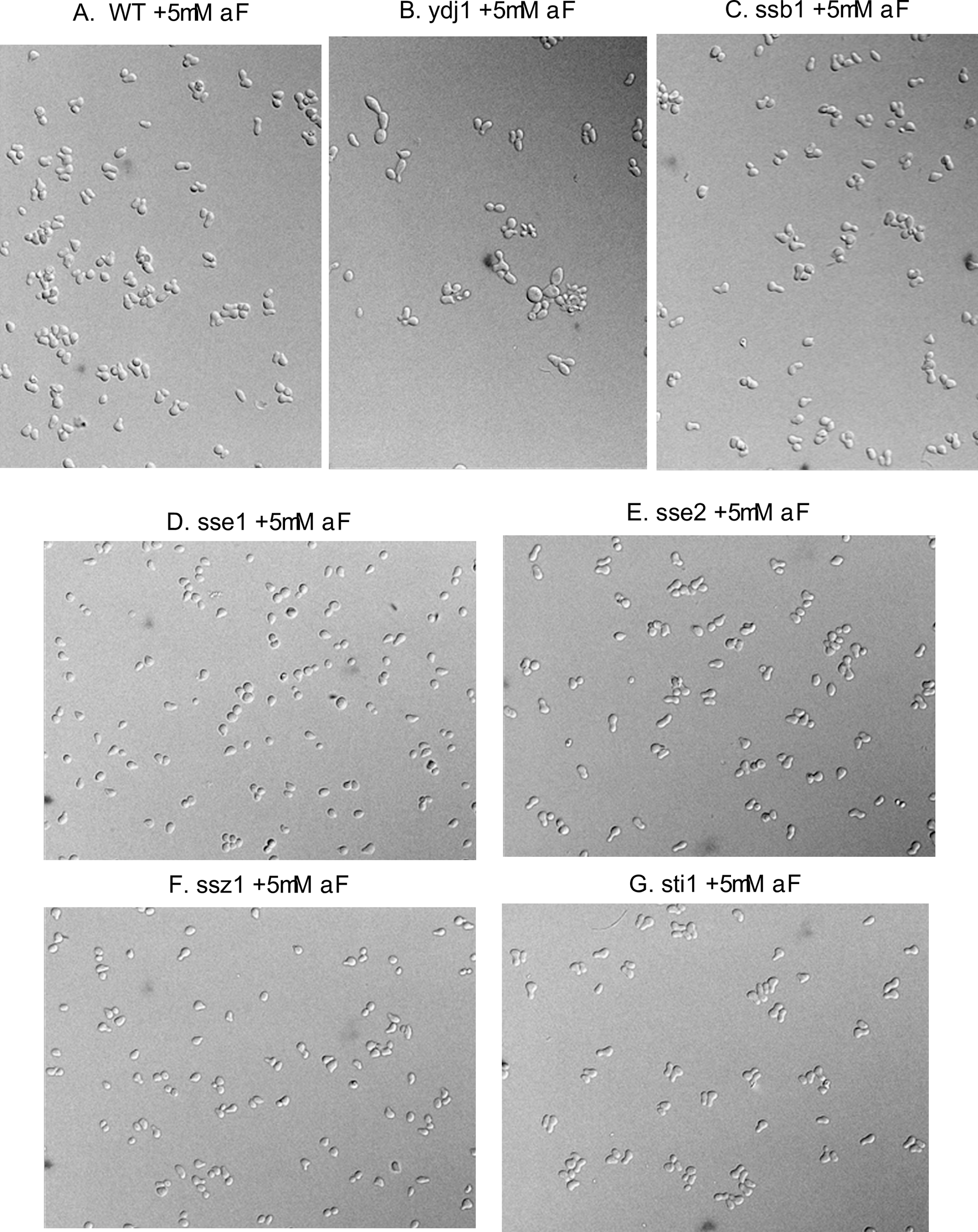

Supplement: S7 Fig — (TIF) [file pone.0289339.s007.tif]

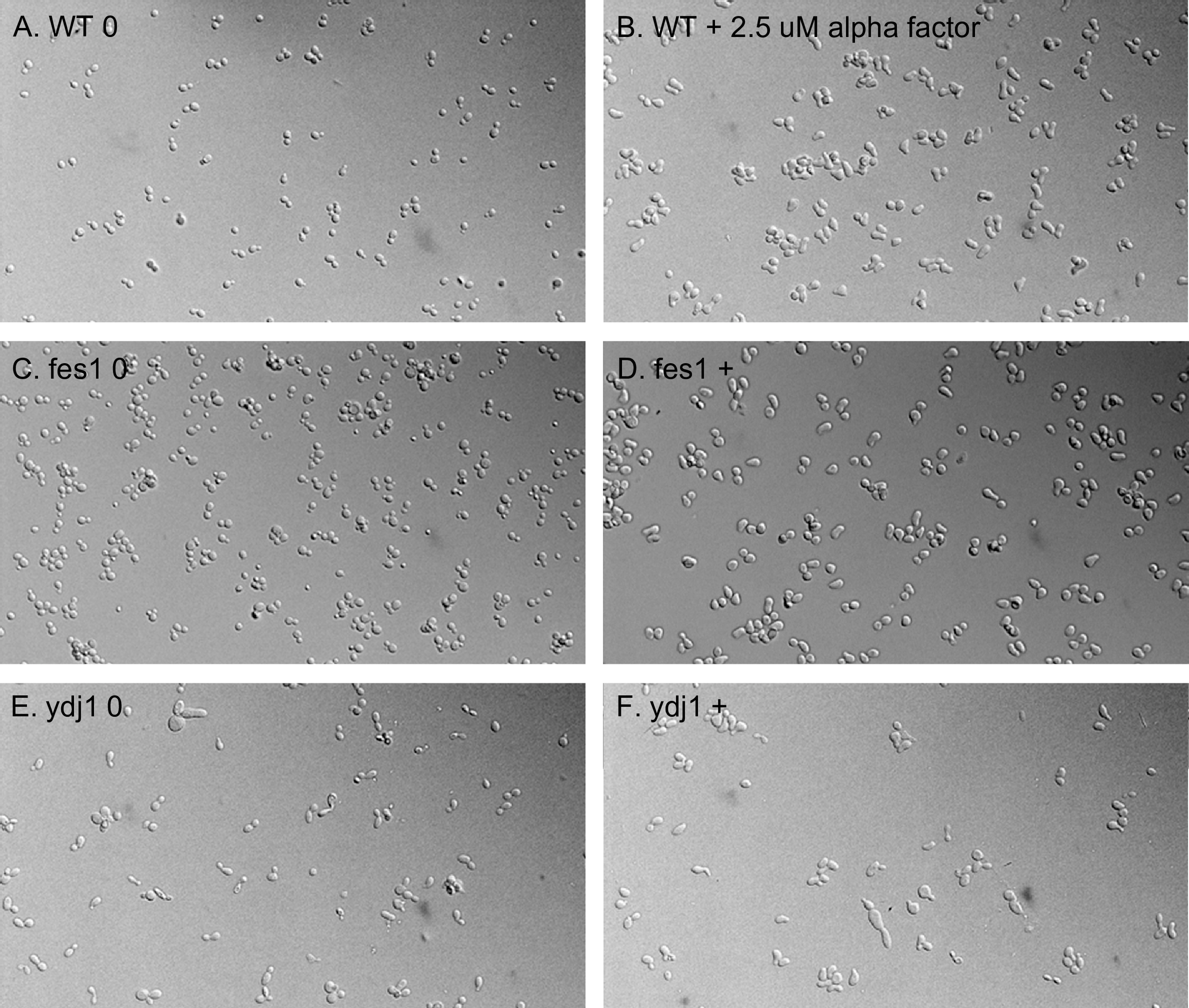

Supplement: S8 Fig — (TIF) [file pone.0289339.s008.tif]

page 1

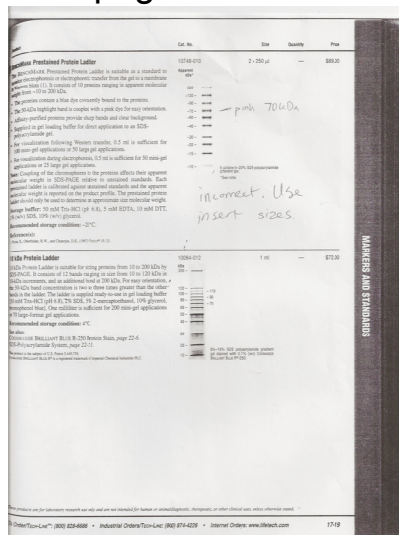

page 2

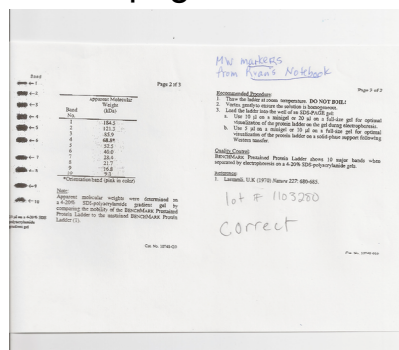

Supplement: S1 Appendix — (PDF) [file pone.0289339.s015.pdf]
