# Supplementary material for: Effects of HSP70 chaperones Ssa1 and Ssa2 on Ste5 scaffold and the mating mitogen-activated protein kinase (MAPK) pathway in Saccharomyces cerevisiae
Source: PLoS One. 2023 Oct 18;18(10):e0289339. doi: 10.1371/journal.pone.0289339 (PMC10584130; doi:10.1371/journal.pone.0289339)
Supplement: S1 File — (DOCX) [file pone.0289339.s016.docx]

**Supporting Information**

**HSP70 chaperones Ssa1 and Ssa2 regulate the Ste5 scaffold and the mating mitogen-activated protein kinase (MAPK) Pathway in *Saccharomyces cerevisiae***

Francis W. Farley, Ryan McCully, Paul B. Maslo, Lu Yu, Mark Andrew Sheff, Homayoun Sadeghi and Elaine A. Elion^*^

*Corresponding author

**Supplemental tables**

**Table S1. Plasmids and strains used in this study.**

**Table S2. Summary of ImageJ densitometry values.**

**Table S3. List of positive and negative regulators of mating and invasive growth proteins screened for interaction with Ssa1, Ssa2 and other HSP70 network proteins in published databases.**

**Table S4. Summary of interaction data on 212 mating pathway and 121 invasive growth pathway proteins with Hsp70 chaperones and co-chaperones.**

**Table S5. Morphology of wild type and *fes1Δ* mutant strains before and after exposure to α factor.**

**Table S6. Cell morphology of Hsp70 family mutants before and after α factor treatment.**

**Supplemental figure titles and legends**

**Figure S1. Analysis of full-length Ste5 (1-917) coding sequence with bioinformatics algorithms.** A. Cartoon of Ste5 with structural domains, domains of interaction with proteins and lipids and dimerization domains. B. PLAAC plot of Ste5. PLAAC (S13, S14) displays the propensity of a protein to adopt a prion or prion-like conformation along with intrinsic unfoldedness shown as the fold index (S15). The regions predicted to be most intrinsically unfolded are underlined in the amino acid sequence below the plot. C. RCS PDB plot (S16) of Ste5. D. Disopred3 analysis of Ste5 (S17). Di shows the amino acid sequence with residues predicted to be disordered enclosed in green boxes. Dii shows a confidence score for disorder (blue line) with regions predicted to interact with protein binding domains (orange line). E. IUPred plot (S18, S19alpred) of disorder tendency for Ste5.

**Figure S2. *ssa1Δ ssa2Δ* double mutant growth at different temperatures.** A. Streakouts of wild-type and *ssa1Δ ssa2Δ* strains gown at room tempierature, 30^o^C and 37^o^C. The *ssa1Δ ssa2Δ* strains did not grow at 37^o^C. B. Viability of a *ssa1::HIS3 ssa2::LEU2 ssa4::*LYS2 triple mutant expressing *SSA1* from a *GAL1* promoter during repression of *SSA1* expression. Strain EYL342 (*ssa1::HIS3 ssa2::LEU2 ssa4::LYS2* + *pGAL1prom-SSA1-URA3-CEN4*) was grown in YEP 2% galactose medium to logarithmic phase, then diluted back and shifted to YEP-2% dextrose medium and aliquots were monitored for viability over time. Viability was measured by plating colonies from the liquid culture onto YEP-2% galactose plates and YEP-2% dextrose plates over the course of a 6 hour shift from galactose medium to dextrose medium. C. EYL342 viability measured by A600.

**Figure S3. Analysis of Ste5ms crystal structures 3FZE and 4F2H overlapping the VWA domain (residues 583-786).** A-E iCn3D renderings of Ste5 crystal structures. A-C. 3FZE ribbon, tube and amino acid side chain views. The 3FZE Ste5ms (residues 593-786) structure is at 1.16 angstrom resolution (S20). The polypeptide backbone is in magenta with the ste5L610/614/634/637A leucine to alanine mutation residues and part of an N-terminal helix are in yellow. D-E. Ribbon and tube views of 4F2H. The 4F2H Ste5(583-786) structure is at 3.18 angstrom (S21). The views show an anti-parallel homodimer with monomers in magenta and blue with the ste5L610/614/634/637A mutation residues and the entire N-terminal helix that dimerizes (residues 583-592) in yellow. F-I. Analysis of amino acid interactions of wild type and mutant Ste5 minimal scaffold dimer 4FH2 amino acid residues 589-798 with iCn3D 3.1.2 at NCBI (S22). Shown are predicted amino acid contacts with the wild type leucine residues and the mutant alanine residues. K. NCBI-BLASTP of *S. cerevisiae* Ste5 minimal scaffold. Positions 610, 614, 634 and 637 in Ste5 are highlighted in grey for identical and pink for similar. I. BIPPRED comparison (S23) of Ste5 wild type versus L610/614/634/637A mutant. Thirteen of the 107 predicted high maximal score (i.e. 0.9-0.99, where 1.0 is a perfect score) for Hsp70 binding in Ste5 have a lower score in the Ste5_L610/614/634/637A mutant than for the Ste5 wild type sequence. They are shown as bars overlapping the mutations. The difference in score between mutant and wild type is shown for 4 potential binding sites.

**Figure S4. Enlarged image and tally of punctate foci in cells expressing ste5L610/614/634/637A with overexpressed Ssa1-GFP.** A. 9E10 localization of Ste5-Myc9 in color. The image shows DAPI staining in blue and ste5L610/614/634/637A-Myc9 in pink. B. Grey scale image of A. C. A key that describes the arrows that indicate some of the many punctate foci and patches visible in the photo. Note that some cells exhibited what appears to be a network of globule like circles of Ste5-Myc9. D. Tallies of cell and inclusion body characteristics.

**Figure S5.** **Comparison of hydrophobicity, bulkiness, average buried and disorder of amino acid residues of wild type Ste5 and mutant Ste5 L610/614/634/637A.** A. Alanine versus leucine characteristics. B-C. Bulkiness plots of Ste5(551-700) and Ste5mut(551-700) using algorithm based on Rose et al. (S25). D-E. Average buried plots on Ste5(551-700) and Ste5mut(551-700) using algorithm based on Zimmerman et al (S26, S27). The wild type 601-660 sequence is 601SLKREKPDN**L** AII**L**QIDFTK LKEEDSLIVV YNS**L**KA**L**TIK FARLQFCFVD RNNYVLDYGS 660 and the mutant sequence is 601SLKREKPDN**A** AII**A**QIDFTK LKEEDSLIVV YNS**A**KA**A**TIK FARLQFCFVD RNNYVLDYGS 660. F. IUPRED plot (S28-S29) of Ste5(551-700). G. IUPRED plot of Ste5L610/614/634/637A (551-700). H. IUPRED plot of known disordered protein that aggregates alpha synuclein. I. IUPRED2 and ANCHOR plots (S30) of Ste5(1-551-700). J. IUPRED2 and ANCHOR plot of Ste5L610/614/634/637A (551-700). Amino acid sequences were analyzed at the IUPRED server and the ISOPRED2 server //iupred2a.elite.hu/ (S28-S30). Other analyses were done at the EXPasy website with default 9 amino acid scanning window (S31).

**Figure S6. Enlarged long and short exposures of Figure 8A anti-Kss1 immunoblot and FUS1::UbiY-lacZ standard error values and p-values.**  A-B. Immunoblot from Figure 5A (A) with a shorter exposure (B). C. Two sets of FUS1::UbiY- lacZ values are shown for wild type (EY3138) and *ssa1Δ ssa2Δ* (EY3139) strains with p-values (students T-test, two independent means, two sided). B. Normalized standard errors are shown for the FUS1-UbiY-lacZ experiments in Figure 8A-B.

**Figure S7. Representative fields of wild type and Hsp70 mutants treated with 2.5 μM α factor.** Cells were grown at room temperature to logarithmic phase and treated with 2.5 μM αF for 90 minutes. A. WT EYL1740 (S288c-BY4741), B. *fes1*, C. *ydj1*. See Table S7 and Table S8 for tallies of morphology.

**Figure S8. Representative fields of wild type and hsp70 mutants treated with 5 μM αF.** Cells were grown at room temperature to logarithmic phase and treated with 5 μM αF for 90 minutes. A. WT EYL1740 (S288c-BY4741), B. *ydj1*, C. *ssb1*, D. *sse1*, E. *sse2*, F. *ssz1*, G. *sti1*. See Table S8 for tallies of morphology.

**S1 Appendix. Benchmark molecular weight standards package insert documentation.**

**Supplemental References**

S1. Lydall D, Ammerer G and Nasmyth K. A new role for MCM1 in yeast: cell cycle regulation of SWI5 transcription. Genes & Dev. 1991; 5: 2405-2419.

S2 Rhodes N, Connell L, Errede B. STE11 is a protein kinase required for cell-type-specific transcription and signal transduction in yeast. *Genes Dev* **1990; 4**: 1862–1874.

S3. Hu Z, Wang Y, Yu L, Mahanty SK, Mendoza NN. and Elion EA. Mapping regions in Ste5 that support Msn5-dependent and –independent nuclear export. Biochem.Cell Biol. 2016; 94: 109-128.

S4. Wang Y and Elion EA. Nuclear export and plasma membrane recruitment of the Ste5 scaffold are coordinated with oligomerization and association with signal transduction components. Mol. Biol. Cell 2003; 14: 2543-2558.

S5. Mahanty SK, Wang Y, Farley FW and Elion EA. Nuclear shuttling of yeast scaffold Ste5 is required for its recruitment to the plasma membrane and activation of the mating MAPK cascade. Cell 1998; 98: 501–512.

S6. Elion EA, Satterberg B and Kranz JE. Fus3 phosphorylates multiple components of the mating signal transduction cascade: Evidence for Ste12 and Far1. Mol. Biol. Cell. 2003; 4: 495-510.

S7. Zhou Z, Gartner A, Cade R, Ammerer G and Errede B. Pheromone-induced signal transduction in *Saccharomyces cerevisiae* requires the sequential function of three protein kinases. Mol. Cell. Biol. 1983; 13(4): 2069-2080.

S8. Toenjes KA, Sawyer MM and Johnson DI. The guanine-nucleotide-exchange factor Cdc24p is targeted to the nucleus and polarized growth sites. Curr. Biol. 1999; 9:1183-1186.

S9. Kranz JE, Satterberg B, and Elion EA. The MAP kinase Fus3 associates with and phosphorylates the upstream signaling component. Ste5. Genes Dev. 1994; 8: 313–327.

S10. Choi KY, Satterberg B, Lyons DM and Elion EA. Ste5 tethers multiple protein kinases in the MAP kinase cascade required for mating in *S. cerevisiae*. Cell 1994; 78:499–512.

S11. Werner-Washburne M, Stone DE and Craig, EA. Complex interactions among members of an essential subfamily of hsp70 genes in Saccharomyces cerevisiae. Mol Cell Biol. 1987; 7:2568-2577.

S12. Certus Technology methods to use N.I.H. ImageJ for densitometry measurements. ImageJ|Sybil <https://www.sybil-fp7.eu/node/95>.

S13. Lancaster AK, Nutter-Upham A, Lindquist S and King OD. PLAAC: a web and command-line application to identify proteins with Prion-Like Amino Acid Composition Bioinformatics 2014; doi:10.1093/bioinformatics/btu310.

S14. Alberti S, Halfmann R, King O, Kapila A and Lindquist S. A systematic survey identifies prions and illuminates sequence features of prionogenic proteins Cell 2009; 137:146–158.

S15. Prilusky J, Felder CE, Zeev-Ben_mordehai T, Rydberg EH, Mann O, Beckmann JS, Silman I and Sussman JL. FoldIndex: a simple tool to predict whether a given protein sequence is intrinsically unfolded. Bioinformatics 2005; 21: 3435–3438.

S16. Berman HM, Westbrook J, Feng Z, Gilliland G, Bhat TN, Weissig H, Shindyalov IN, Bourne PE. The Protein Data Bank. Nucleic Acids Research 2000; 28: 235-242. doi:10.1093/nar/28.1.235 URL rcsb.org

S17. Ward JJ, Sodhi JS, McGuffin LJ, Buxton BF and Jones DT. Prediction and functional analysis of native disorder in proteins from the three kingdoms of life. J. Mol. Biol. 2004; 337: 635-645. DISOPRED2, DISOPRED3 server at http://bioinfDOTcsDOTuclDOTacDOTuk/psipred/

S18. Dosztányi Z, CsizmokV, Tompa P and Simon I. IUPred: web server for the prediction of intrinsically unstructured regions of proteins based on estimated energy content Bioinformatics Applications Note 2005. 21: 3433–3434.

S19. Dosztanyi Z. Prediction of protein disorder based on IUPred. Protein Science 2017; 27: 331-340.

S20. Good M, Tang G, Singleton J, Remenyi A and Lim, WA. The Ste5 scaffold directs mating signaling by catalytically unlocking the Fus3 MAP kinase for activation. Cell 2009; 136: 1085–1097.

S21. Zalatan JG, Coyle SM, Rajan S, Sidhu SS, and Lim WA. Conformational control of the Ste5 scaffold protein insulates against MAP kinase misactivation. Science 2012; 337: 1218–1222.

S22. Wang J, Youkharibache P, Marchler-Bauer A, Lanczyckj C, Zhang D *et al*. iCn3D: From Web-Based 3D Viewer to Structural Analysis Tool in Batch Mode. Front. Mol. Biosci. 2022 9: 831740.

S23. Schneider M, Rosam M, Glaser M, Patronov A, Shah H, Back C, Daake A, Buchner J and Antes I. BiPPred: Combined sequence- and structure-based prediction of peptide binding to the Hsp70 chaperone BiP. Proteins Structure Function Bioinformatics. 2016; 84:1390-1407.

S24. Slabinski, L, Jaroszewski, L., Rychlewski, L, Wilson, IA, Lesley, SA and Godzik, A. XtalPred: a web server for prediction of protein crystallization. Bioninformatics 2007; 23: 3403-3405. XTalPred-RF server at.http://ffasDOTburnhamDOTorg/XtalPred.

S25. Rose GD, Geselowitz AR, Lesser GJ, Lee RH, and Zehfus MH. Hydrophobicity of amino acid residues in globular proteins. Science. 1985;229:834-838.

S26. Zimmerman JM, Eliezer N and Simha R. The characterization of amino acid sequences in proteins by statistical methods. Journal of Theoretical Biology 1968; 21:170-201.

S27. Jahandideh S, Jaroszewski L and Godzik A. Improving the chances of successful protein str5cture determination with a random forest classifier 2014; Acta Crystallog.r D. Biol. Crystallogr. 70(Pt 3),627-663.

S28. Dosztányi Z, CsizmokV, Tompa P and Simon I. IUPred: web server for the prediction of intrinsically unstructured regions of proteins based on estimated energy content Bioinformatics Applications Note 2005. 21: 3433–3434.

S29. Dosztanyi Z. Prediction of protein disorder based on IUPred. Protein Science 2017; 27: 331-340.

S30. Meszaros B, Erdos G and Dosztanyi Z. IUPred2A: context-dependent prediction of protein disorder as a function of redox state and protein binding. Nucl. Acids Res 2018; 46: W329-W337.

S31. Gasteiger E, Hoogland C, Gattiker A, Duvaud S, Wilkins MR, Appel RD and Bairoch A. Protein Identification and Analysis Tools on the ExPASy Server (In) John M. Walker (ed): The Proteomics Protocols Handbook, Humana Press 2005; pp. 571-607 web.expasy.org

S32. Gong Y, Kakihara Y, Krogan Y, Greenblatt J, Emili E, Zhang Z= and Houry WA. An atlas of chaperone–protein interactions in *Saccharomyces cerevisiae*: implications to protein folding pathways in the cell Molecular Systems Biology 2009; 5: 275-289.

S33. Truman AW, Kristjansdottir K, Wolfgeher D, Hasin N, Polier S and Zhang H. CDK-Dependent Hsp70 Phosphorylation Controls G1 Cyclin Abundance and Cell-Cycle Progression. Cell 2012; 151: 1308–1318.

S34. Cherry J, Hong EL, Amundsen C, Balakrishnan R, Binkley G, Chan ET, Christie KR, Constanzo MC *et al.* Saccharomyces Genome Database: the genomics resource of budding yeast. Nucleic Acids Res.2012; 40: D700–D705.

S35. Orchard, S. Ammari, M., Aranda, B., Breuza, L., Briganti, L., Broackes-Carter, F. et al. The MI project—IntAct as a common curation platform for 11 molecular interaction databases. Nucleic Acids Res. 2014; 42(D1): D358-D363.

S36. Babu, M, Vlasblom, J, Pu, S., Guo, X, Graham, C, Bean, BDM, Burston, H E, Vizeacoumar, FJ, Snider, J, Phanse, S, Fong, V, Yi, Y, Tam, C, Davey, M, Hnatshak, O, Bajaj, N, Chandran, S, Punna, T., Christopolous, C, Wong, V, Yu, A, Zhong, G, Li, J, Stagljar, I, Conibear, E, Wodak, SJ, Emili, A., Greenblatt, JF. Interaction landscape of membrane-protein complexes in *Saccharomyces cerevisiae* Nature 2009; 489: 585-590.

S37. Willmund, F, del Alamo, M, Pechmann, S, Chen,T, Albanese,V, Dammer, EB, Peng, J, and Frydman, J. The Cotranslational Function of Ribosome-Associated Hsp70 in Eukaryotic Protein Homeostasis. Cell 2013; 152: 196–209.

S38. Jamuczak AF, Evers CE, Schwartz J-M, Grant CM and Hubbard SJ. Quantitative proteomics and network analysis of SSA1 and SSB1 deletion mutants reveals robustness of chaperone HSP70 network in Saccharomyces cerevisiae. Proteomics. 2015; 15: 3126-3139.

S39. Orchard S, Ammari M, Aranda B, Breuza L, Briganti L, Broackes-Carter F, *et al.* The MI project—IntAct as a common curation platform for 11 molecular interaction databases. Nucleic Acids Res. 2014; 42(D1): D358-D363.

S40. Wiederhold E, Veenhoff LM, Poolman B, and Slotboom J. Proteomics of *Saccharomyces cerevisiae* organelles. Mol. And Cell. Proteomics. 2010; 9.3: 431-445.

S41. Sharma K, Vabulas RM, Macek B, Pinkert S, Cox J, Mann M et al. Quantitative proteomics reveals that Hsp90 inhibition preferentially targets kinases and the DNA damage response. Mol Cell Proteomics. 2012; 11 [M111 014654]

S42. Zhao R, Davey M, Hsu YC, Kaplanek P, Tong A, Parsons AB et al. Navigating the chaperone network: an integrative map of physical and genetic interactions mediated by the hsp90 chaperone. Cell 2005; 120: 715-727.
